# Editorial: Refining Prevention: Genetic and Epigenetic Contributions

**DOI:** 10.3389/fpsyg.2016.00145

**Published:** 2016-02-11

**Authors:** Steven R. H. Beach, Jessica M. Sales

**Affiliations:** Center for Family Research, University of GeorgiaAthens, GA, USA

**Keywords:** prevention, translation, genetics, epigenetics, substance use, mental health

The current series of articles was designed to capture ongoing translational activities linking genetic and epigenetic research to enhancement of prevention and treatment efforts. Better understanding the processes associated with better and worse response to a range of environmental causes and to preventive interventions is a critical first step in refining and adapting existing prevention programs, or alternatively designing new prevention programs with enhanced outcomes. In the current series of papers we address translational issues from several directions. Our first three papers highlight methodological and conceptual innovations with the potential to guide researchers in new directions and maximize the impact of ongoing G and GxE research. The next set of four manuscripts provide an excellent example of informative empirical tests of G and GxE effects on key symptoms, mediators, and outcomes. This type of work highlights the potential for genetically informed research to illuminate variable base rates of target behavior, build upon GWAS results to test specific, theory driven hypotheses, and highlight populations with greater sensitivity to common environmental stressors. Our last two contributions point to future directions, the first examining innovative strategies that may be transportable to many contexts, allowing researchers to develop broad coalitions to examine genetic effects on behavioral health, and the second introducing epigenetic variables as a way to further inform preventive research and illustrate the key role of family environments in laying the groundwork for young adult health. Together the series provides a useful overview of strategies and approaches as well as important insights for future translational efforts. To guide examination of specific manuscripts, we briefly characterize each below.

The first paper by Howe and colleagues describes a new research design and analytic approach, the baseline target moderated mediation (BTMM) design, and characterizes its utility for enhancing the investigation of genotypic moderators of prevention trial results, and strengthen the potential for such investigations to lead directly to treatment innovation. Ultimately, this approach will help researchers more readily identify subgroups most likely to respond, help explicate the mechanisms involved, and sidestep many of the potential difficulties associated with genetic selection in primary prevention.

Our second paper by Philibert and colleagues introduces a new epigenetic measurement tool to advance the examination of preventive intervention efficacy as well as treatment efficacy in the context of smoking reduction and prevention. They identify methylation status at a CpG locus in the aryl hydrocarbon receptor repressor, cg05575921, as a sensitive and specific indicator of smoking status and history in young adults, suggesting that utilization of this locus in a methylation-based diagnostic scheme could be a great help in documenting prevention effects and improving the characterization of response to treatment of smoking, and smoking related disorders.

Our third paper by Gray and MacKillop provides an overview of the literature on delayed reward discounting (DRD), an index of how much an individual devalues a future reward based on its delay in time. This novel construct is explicated in part by examination of genetic and environmental correlates. Given its robust association with various drugs of abuse, DRD appears to be worthy of investigation at a more general level as a novel and promising drug abuse prevention target.

The second section of our series begins with a paper by Obasi and colleagues, who find that perceived stress and alcohol consumption have a deleterious effect on HPA-axis functioning. In particular, perceived stress and alcohol consumption disrupt the cortisol awakening response, an effect that is superimposed on genetic influences. Likewise, the next paper in this section by Nikolova and colleagues uses a sophisticated mix of methods to examine the effect of a SNP previously linked to depression by GWAS investigation. They show that that carrying the A allele of *FREM3* may be implicated in blunted amygdala reactivity, slower reaction times in face-matching, and marginally slower performance on the Tail Making Test, suggesting possible brain mechanisms linking genotype to outcomes. Our sixth paper by Windle and Mrug uses a GxE approach to explicate the role of disrupted attachment on depression. In particular, they show that young adult females who experienced parental divorce during adolescence and have the “GG” oxytocin genotype had substantially greater risk of depressive symptoms compared to those experiencing parental divorce but carrying an “AA” or “AG” genotype. Our seventh paper by Sales and colleagues explores the effect of carrying the “*s*” allele at the 5HTTLPR, and its effect on increased reactivity to experiences of discrimination, experiences that are all too common among young adult African-Americans. They find that high levels of racial discrimination are significantly associated with greater odds of high depressive symptoms only for participants with the “*s*” allele, with an interaction predicting depressive symptoms among African–American adolescent females.

Our eighth paper in the series by Dick and Hancock describes the “Spit for Science” project, a remarkably successful project conducted at a large, public, urban university in the United States that was able to unify efforts across campus to address problematic college student substance use and mental health issues, creating large new data sources for examining genetic contributions to behavioral health outcomes in the process. This program provides a template for the type of creative scientific paradigms that may be useful, and indeed essential, if efforts to personalize behavioral health interventions are to move forward.

The ninth and final paper by Beach and colleagues illustrates the potential power of the “epigenetic” measurement tools that have recently become available to family and prevention science researchers. These tools provide an opportunity to identify potential biological mechanisms linking family contexts and health or health behavior outcomes. This paper suggests that examination of epigenetic mechanisms offer considerable promise for family researchers to further expand their etiological models. In particular, the authors find that epigenetic regulation of a key inflammatory factor, Tumor necrosis factor (TNF) is associated with early family environment, and mediates the association of earlier parenting with young adult health outcomes years later.

## Author contributions

All authors listed, have made substantial, direct and intellectual contribution to the work, and approved it for publication.

## Funding

Both authors and this research topic are supported by Grant P30DA027827 from the National Institute on Drug Abuse.

### Conflict of interest statement

The authors declare that the research was conducted in the absence of any commercial or financial relationships that could be construed as a potential conflict of interest.

